# A Cognitive Mobile BTS Solution with Software-Defined Radioelectric Sensing

**DOI:** 10.3390/s130202051

**Published:** 2013-02-05

**Authors:** Jorge Muñoz, Javier Vales Alonso, Francisco Quiñoy García, Secundino Costas, Marcos Pillado, Francisco Javier González Castaño, Manuel Garćia Sánchez, Roberto López Valcarce, Cristina López Bravo

**Affiliations:** 1 Gradiant, CITEXVI, Campus Universitario, Vigo 36310, Spain; E-Mail: jorgem@gradiant.org; 2 Department of Communications and Information Technologies, Universidad Politécnica de Cartagena, Cartagena 30202, Spain; E-Mail: javier.vales@upct.es; 3 AtlantTIC, University of Vigo, Campus Universitario, Vigo 36310, Spain; E-Mails: javier@gti.uvigo.es (F.Q.G.); secunc@gts.tsc.uvigo.es (S.C.); mpillado@gti.uvigo.es (M.P.); manuel.garciasanchez@uvigo.es (M.G.S.); valcarce@gts.tsc.uvigo.es (R.L.V); clbravo@det.uvigo.es (C.L.B.)

**Keywords:** SDR, cognitive radio, mobile communications, vehicular communications, BTS, cellular communications

## Abstract

Private communications inside large vehicles such as ships may be effectively provided using standard cellular systems. In this paper we propose a new solution based on software-defined radio with electromagnetic sensing support. Software-defined radio allows low-cost developments and, potentially, added-value services not available in commercial cellular networks. The platform of reference, OpenBTS, only supports single-channel cells. Our proposal, however, has the ability of changing BTS channel frequency without disrupting ongoing communications. This ability should be mandatory in vehicular environments, where neighbouring cell configurations may change rapidly, so a moving cell must be reconfigured in real-time to avoid interferences. Full details about frequency occupancy sensing and the channel reselection procedure are provided in this paper. Moreover, a procedure for fast terminal detection is proposed. This may be decisive in emergency situations, e.g., if someone falls overboard. Different tests confirm the feasibility of our proposal and its compatibility with commercial GSM terminals.

## 1. Introduction

Nowadays different cellular systems support worldwide wireless communications. The fusion of these systems with Internet provides flexible data services, enabling a new wave of mobile applications beyond traditional voice/message services. This great extent of flexibility at the upper communication layers contrast with the static and fixed core physical layers. Although in most cases this model is enough to satisfy users' needs, the use of flexible radios may offer new options and applications, thanks to real-time reconfiguration of radio resources.

In this paper we introduce a new concept for communications inside vehicles based on software defined radio: a vehicular cell that is able to auto-reconfigure its operation channel to avoid interferences to/from neighbor cells. This cell provides both traditional services (voice/messages) and innovative ones such as user real-time detection.

This mobile cell can be used in any large vehicle whose internal communications could be provided by means of a cell. There are no additional requirements to its application domain. Some illustrative examples are ships or trains.

Vehicular communications involve data exchange within and between vehicles and between vehicles and infrastructure elements. Internal communications are especially useful in large vehicles, such as ships, where they may help to enforce safety. In contrast to cellular systems, VHF walkie-talkies indoor coverage is low. Although these systems operate at a lower frequency than second or third generation cellular systems, the higher sensitivity and transmission power of base station equipments, as well as the shorter and better communication paths, allow for better coverage in cellular systems. In [1], the authors conclude that broadband VHF signals inside vessels may not be feasible due to the rich multipath environment created by metallic structures, whereas in [2] the authors show that communication through ship compartments, despite these same effects, is possible in the frequency range of 0.8–2.5 GHz, where the cellular systems operate.

In addition, VHF devices, mandatory in most vessels, are not allowed for personal communications. The main reasons are that VHF radiotelephones could interfere with safety-related VHF systems and that VHF frequencies are not suitable for communications in indoor locations. Therefore, a new technology for internal communications would enjoy high demand but, for outperforming current solutions, it should combine the following features:

- **Wireless technology** (allowing ubiquitous communications within the vessel).
- **Deployment simplicity** and **compatibility with inexpensive commercial terminals**.
- **Low cost**, resulting from using off-the-shelf commercial components.
- **Interference-free communication**, in the sense that it should not interfere with nearby in-shore communication systems or those of other vessels.

The Global System Mobile (GSM) [3] is a well-known 2G technology which satisfies the first two requirements, and Software-Defined Radio (SDR), which allows a given physical system to take different operational roles along the time by reusing hardware, can meet the other two.

First, SDR allows the implementation of low-cost mobile private Base Transceiver Stations (BTSs) as well as the switching functionalities required to enable communications in every ship. Although maritime wireless communications have motivated intense research [4–8], they focus on ship-to-ship and ship-to-shore communications. A low-cost GSM solution based on SDR has not been proposed for internal ship communications so far. Second, SDR flexibility allows the implementation of a Cognitive Radio (CR) module with spectrum sensing capabilities to avoid busy frequencies as the ship approaches the coast or other vessels, as we propose in this paper.

Cognitive Radio (CR) [9,10] relies on sensors to detect activity in the bands of operation. One of its main benefits is spectrum sharing. There are many licensed bands for different radio technologies, but many of them are heavily underutilized. One possible way to optimization is to allow unlicensed users to opportunistically employ licensed frequencies when they are idle. CR manages this and benefits from SDR technology: it is possible to develop context-dependent systems, optimize battery life, design collaborative transmission protocols, and so forth.

**Figure 1.**
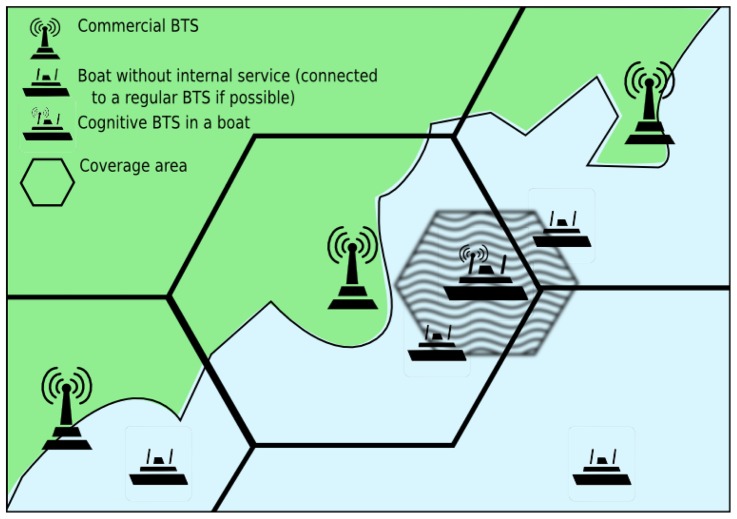
System overview.

In this paper we propose, develop and test the concept of low-cost *cognitive mobile BTS*. On the one hand, our proposal provides normal BTS communications, whereas, on the other hand, it provides cognitive sensing features to prevent interferences with coastal BTSs and other vehicles. Figure 1 depicts a possible scenario for our system: a private BTS on board a ship reselects channels to avoid interfering with coastal BTSs.

We exploit one of the key properties of SDR, its flexibility, to reconfigure the basic hardware to support the sequence of operational modes (e.g., sensing → transmission). A novel dynamic channel change mechanism handles ongoing communications seamlessly. In addition, as SDR confers the possibility to develop new value-added services, we have included a special functionality for terminal detection in emergency situations. In a few seconds, it allows the BTS to identify all the terminals in its area of influence, and consequently to detect that a person has fallen overboard, for example. This functionality could be added to a healthcare system with a more general purpose, like the one described [11]. It is important to remark that the user terminals in our system are normal commercial devices, and therefore it can be deployed at low cost.

In the rest of this paper we review the background (Section 2) and describe the details of its architecture and implementation (Section 3). In Section 4 we validate it with real tests and demonstrate its suitability for the target scenarios. In Section 5 we conclude the paper.

## 2. Related Work

Traditional radio systems rely on hardware elements (HDR, Hardware Defined Radio), which are constrained to the fixed characteristics of the modules performing the radio functions. In SDR systems however, certain functions are implemented as modifiable software [12], which confers flexibility, and thus important advantages:

- New products reach the market sooner.
- Software reuse reduces development costs.
- New features can be easily added to existing infrastructures.
- Logistics are simplified thanks to the use of the same platform for different markets.

Some works such as [13] have proposed HDR systems for private communications inside large vehicles. However, their complexity has prevented a real implementation in most cases.

SDR, on the contrary, is progressively drawing interest as a viable option for real implementations of mobile devices. Seo *et al.* [14], for example, presented a GPS SDR sensor with adaptive beam-steering capability for anti-jam applications, and Ryu *et al.* [15] showed that SDR can be applied in true commercial BTS subsystems in a study in which they addressed the design of the hardware and software architectures of a smart antenna base station (SABS) operating in a cellular network.

From a more general perspective, Ramos and Madani [16] proposed a reconfigurable mobile architecture and identified the technologies needed to support reconfigurability. The architecture has intelligence to evolve according to changes in the network. In our work, the cognitive BTS actually follows this philosophy, as part of an intelligent serving mobile network.

Anand *et al.* proposed VillageCell [17], a low-cost, SDR open-source solution to provide free local and long-distance communications to remote regions. VillageCell is based on a GSM cellular network in conjunction with a local rural-area network for VoIP services, which are implemented using OpenBTS and Asterisk. However, unlike us, the authors did not consider channel interference sensing or handover, two scenarios for which we provide solutions.

Several GSM-based safety systems have been already proposed for terminal detection. For example, Mondin *et al.* [18] introduced the Helios Platform, an unmanned aerial vehicle used as a mobile base station. It is a cost-effective solution for covering low-user-density, impervious, or offshore locations, which minimizes interferences with an adaptive beam-forming smart antenna. However, it has no provision for cognitive spectrum reuse. Besides, if a rapid response were necessary (e.g., if a crew member fell overboard) the deployment time of Helios would be prohibitive, unless a communication system such as the one we propose was available between the vessel and the life jacket. Wypych *et al.* [19] described AirGSM, a similar system with the same limitations. Zorn *et al.* [20], in turn, described a search-and-rescue system that combines a jammer with an SDR BTS unit to disable normal communications and force terminals to register themselves with the new BTS, a relatively old concept [21]. However, there is no provision for cognitive channel allocation.

Finally, we must remark that part of our work relies on the OpenBTS project [22], in conjunction with the Universal Software Radio Peripheral (USRP) [23]. Although several research works have employed this configuration (e.g., [24–28]), as far as we know none of them have implemented automatic channel selection or seamless migration between old and new channels.

## 3. Cognitive Mobile BTS

**Figure 2.**
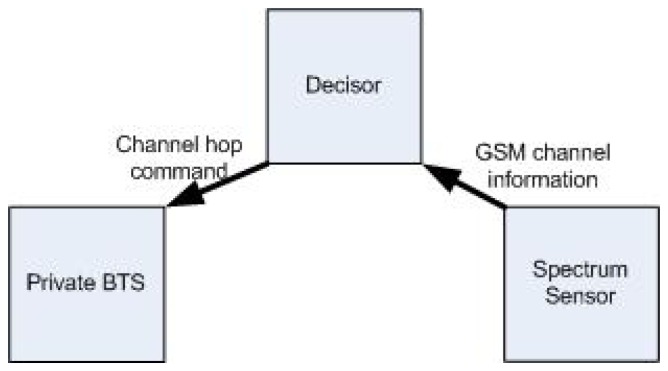
Cognitive BTS modules.

Our private cognitive BTS consists of the three modules shown in Figure 2: (i) Private BTS, (ii) Spectrum Sensor Module (SSM) and (iii) Decision Module (DM).

The private BTS module is the core element, comprising both the radio transceiver (*i.e.*, the module implementing the GSM radio interface) and the switchboard functionalities allowing communications between registered Mobile Stations (MSs). As an additional functionality, it provides controlled channel change, that is, the ability of migrating current communications to a new frequency channel.

The SSM also performs continuous spectrum sensing in order to detect used and empty spectrum channels. This information is passed to the DM, which is in charge of deciding when a channel change is necessary. Decision criteria must prevent communication degradation. One possible mechanism could be periodic change to an idle channel, but this would not ensure interference-free communications if the period was too long. Moreover, it would force the MSs to continuous channel changes if the period was too short, draining their batteries and reducing communications capacity during these periods. Therefore, the criterion is based on an estimation of the quality of communication in the current channel, similarly to what occurs with conventional GSM traffic channel changes. In other words, when high degradation is detected, the communication is transferred to a suitable new channel selected by the DM. The following procedures have been implemented:

- Downlink channel control: allows to detect interferences affecting each particular downlink connection, it is based on the measurement reports received from each MSs, like in conventional GSM networks.
- Uplink channel control: it is the counterpart of the previous procedure, allowing interference control for the uplink connections. It is performed by obtaining quality measurements of the channel, like conventional GSM networks.
- Idle mode control: permits detecting interferences while no active connection exists between the BTS and the MS. This control procedure is unavailable in conventional GSM networks, since in them MSs will tune to some neighbor cell of the same network to avoid the interference source. In our case, there is a single cell, and therefore the normal control procedure is not possible. We propose the following mechanism to minimize the impact of interferences in case of idle MSs: (i) force periodic communications from the MSs to the cognitive BTS and (ii) perform channel reselection if a given ratio of previously registered MSs do not transmit periodic messages.

When the switchboard receives the change command from the DM, it notifies to all registered MSs of the newly selected channel. As we will explain later, channel changes are transparent to ongoing communications. This is a highly relevant feature for an SDR-based mobile BTS implementation in which just one cell is operative at any given time.

**Figure 3.**
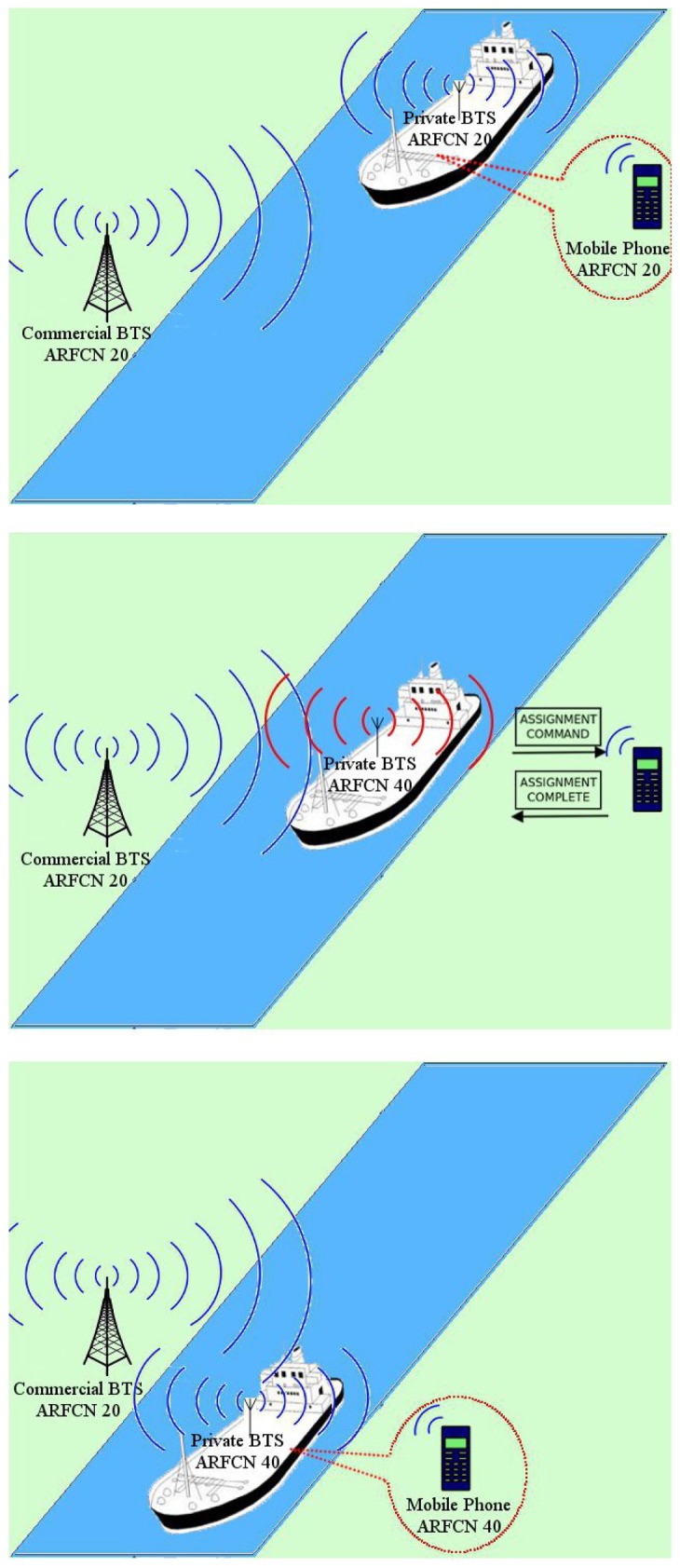
System behavior.

The cognitive capability avoids possible interferences caused by other systems operating in the same channel, as it is illustrated in Figure 3. The private BTS deployed in a ship gives coverage to crew terminals. Once the system sensors detect another signal in the current operating channel, a new channel is activated and communications are redirected to it. A standard cargo ship travels at around 25 knots [29], *i.e.*, 46.3 km·h^−1^. The GSM standard is designed to tolerate speeds of 250 km·h^−1^, so the Doppler effect is not relevant in our scenario, in which the users travel with the BTS inside the ship.

An additional service, which is not available in commercial GSM networks, is the fast identification of the MSs that are reachable at a given moment by performing controlled channel changes: in them, active terminals will automatically respond, allowing the system to identify and potentially locate them quickly in case of emergencies.

In-depth implementation details are provided in the next sections.

### 3.1. Spectrum Sensor Module

The SSM explores the frequency bands of interest (the GSM bands) to detect active radio-frequency transmissions. In GSM communications, each band is duplexed into two different sub-bands, for uplink (phone to BTS) and downlink (BTS to phone) traffic. The BTS transmits control information in the downlink, which is in turn subdivided into different channels, each identified by an ARFCN (Absolute Radio Frequency Channel Number). The number of channels varies depending on the GSM band. For example, GSM1800 has 374 channels (512 to 885) and GSM900 has 124 (1 to 124).

Several possibilities for spectrum sensing are described in the literature [30], such as energy detection sensing, waveform-oriented sensing, cyclostationarity-based sensing, radio technology identification-based sensing, multitaper spectral estimation, wavelet transform-based estimation, Hough transform or time-frequency analysis. For our approach we have chosen energy detection based on periodogram computation, since it is a low complex method that can be easily implemented on SDR systems, and has been reported previously to effectively sense GSM spectrum [31].

Thus, the SSM provides accurate information about activity detected in downlink channels (*i.e.*, in each ARFCN), tagging them as busy or idle, in order to select candidate channels for communications. In the next step, the resulting information (a list sorted by channel energy level) is provided to the DM.

**Figure 4.**
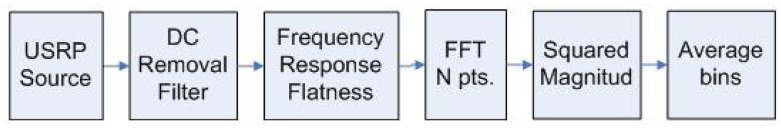
SSM, block diagram.

The SSM consists of the following sequence of processing blocks (Figure 4):

1. USRP source: provides I/Q baseband samples from the band of interest.
2. DC removal filter: removes undesired DC signals generated by the USRP.
3. Frequency response flatness processing: equalizes the incoming signal.
4. *N*-point FFT: calculates the *N*-point Fast Fourier Transform of the incoming data.
5. Squared magnitude: calculates an estimate of bins power.
6. Bin average: averages successive bin blocks of GSM channels.

Once the DM is launched, it activates the SSM, which senses the spectrum. The number of bins of the Fast Fourier Transform (FFT) block determines sensing resolution. The maximum sensed bandwidth per snapshot will generally depend on hardware capacity, and will typically be less than the total target bandwidth. For example, in order to cover the whole GSM900 downlink, the DM must launch the SSM several times, changing the center frequency and storing the result for all the corresponding sub-bands, until the whole 25 MHz bandwidth has been swept. Once the complete band of interest has been scanned, the DM stops the SSM and the collected information is processed. The different bins are grouped by ARFCN. From each group, the system estimates the average power of each GSM downlink channel. When the sensing stage is complete, the detected carriers are sorted by measured power.

### 3.2. Decision Module

**Figure 5.**
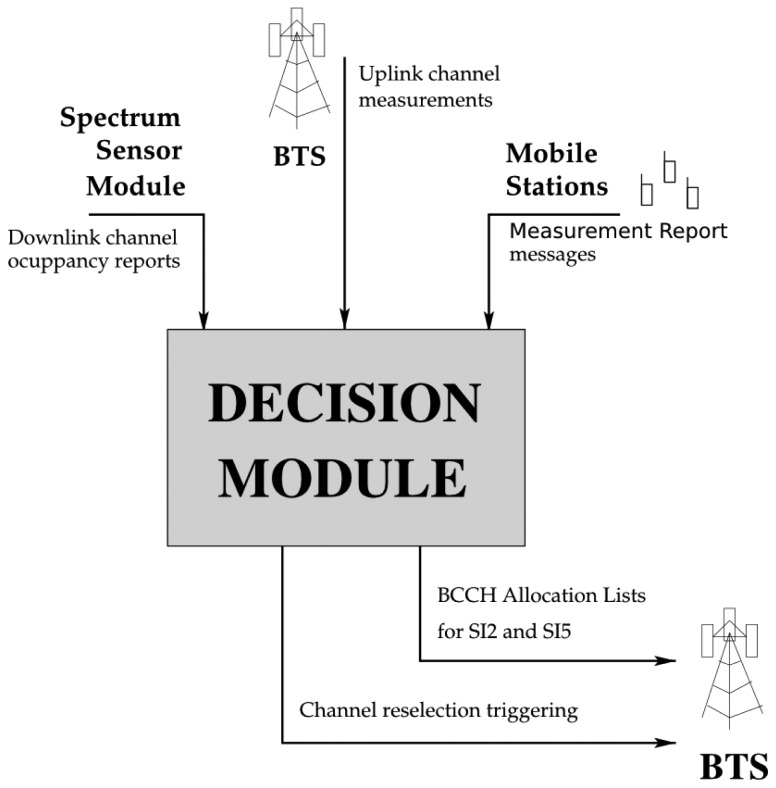
Decision Module.

Figure 5 shows the basic workflow of the Decision Module (DM). As previously stated, the DM is in charge of triggering the channel reselection procedure. Three different sources of information support this decision:

1. The downlink channel occupancy reports of the SSM.
2. The measurement reports provided by the MSs in dedicated mode.
3. The uplink channel measurements of the BTS.

### 3.3. Downlink Channel Control

**Figure 6.**
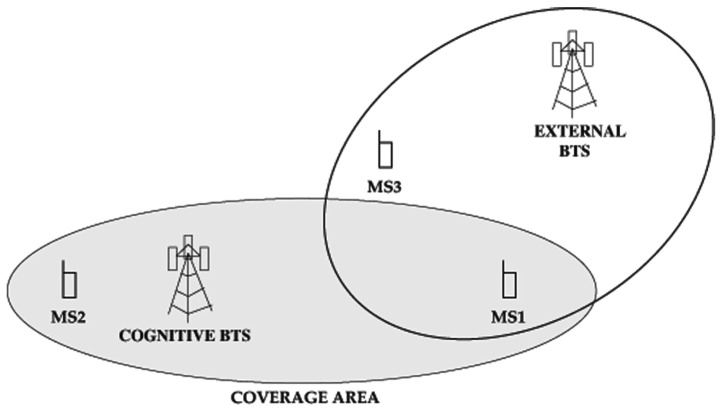
Hidden node phenomenon.

Although the SSM provides information about downlink channel usage, this information is incomplete. MSs may experience hidden node phenomena, just like any wireless system. This situation is represented in Figure 6. The external BTS interferes with MS1, but the SSM is unaware of this (note that the cognitive BTS selects channels statistically, rather than following a plan as in conventional networks, meaning that hidden BTSs are possible). Besides, even if the external BTS was not hidden to the cognitive BTS, the received power levels could differ drastically from those received by the MSs, and these effects would prevent good perception of downlink channel status as sensed by the MSs. The solution thus is to use information obtained by the MSs themselves.

According to the GSM standard [32], MSs with ongoing communications (in Radio Resource connected mode) send Measurement Report messages regularly to the network. These messages contain measurement results about reception characteristics from the current cell and from neighbour cells. The BCCH Allocation list, which is the reference for the measurements, is compiled from information received on the BCCH in System Information 2 and on the SACCH in System Information 5 messages. If neighboring cell information is not available for the serving cell, the mobile station indicates this in a Measurement Report message, which is sent on the link layer Slow Access Control Channel (SACCH). These messages report to the network measurement results about the dedicated channel and the neighbour cells [33].

Channel reselection takes place when the received signal quality level (for any ongoing communication) drops below a minimum RX-Qual (RX-Qual indicates the corresponding Bit Error Rate (%) for the measured channel) threshold (we selected the minimum 9 dB *C*/*I* ratio for GSM communications [34]) or when the periodic monitoring message is not received (note that SACCH messages are always transmitted in confirmed mode).

In order to select a new channel, and since the information provided by the SSM may be insufficient as discussed above, the channel measurements from the MSs are also taken into account. In this case, the parameter considered from the Measurement Reports is the RX-Level (the spectral power detected in the channel) of neighbouring cells, since we want to avoid non-GSM radio sources as well. The cognitive BTS selects candidate cells by considering the four channels where the SSM senses the lowest power. Then, the estimation is completed by jointly averaging the power levels received by the MSs and the perceived power level reported by the SSM. Finally, the cell with minimum detected power is selected for the cell reselection. The rationale behind considering SSM perceiver power level as well is that new MSs might become active, and since the number of MSs with ongoing communications may be low, the SSM represents an average view of the channel. Figure 6 shows this: MS2 may become active and its channel perception would be very different from that of MS1.

### 3.4. Uplink Channel Control

The uplink channel may also experience the hidden node phenomenon. For example, in Figure 6, MS3 may be blocking the uplink channel of MS2, but not the downlink channel. Therefore, the cognitive BTS must use information about signal quality in the uplink. Reference [32] describes how quality measurements are performed in the uplink. They are calculated on each SACCH multi-frame whenever a dedicated connection exists, associated with either a Traffic Channel (TCH) or a Standalone Dedicated Control Channel (SDCCH). The reported parameter (Rx-Qual) is the received signal quality, averaged over the reporting period of an SACCH multi-frame, defined in [33]. As in the downlink, we considered a *C*/*I* ratio of 9 dB. If signal quality drops below this threshold for any active communication, the cell reselection procedure is performed as described above.

### 3.5. Idle Mode Control

If there are no ongoing communications between the MSs and the BTS, the procedure will rely exclusively on information available at the cognitive BTS. Therefore, cell reselection will be based on the channel occupancy reports from the SSM.

In this case, if an interfering source is affecting the downlink, MSs communications may end with a radio link failure. A downlink signaling failure takes place after the expiration of the signaling failure counter *DSC* [33]. When the MS camps on a cell, it is initialized to ⌊90/*N* + 0.5⌋, where *N* is the value of the BS_PA_MFRMS cell parameter [35]. Later, if a paging message is successfully decoded, *DSC* ← *min*(*DSC* + 1, ⌊90/*N* + 0.5⌋), otherwise *DSC* ← *DSC* − 4. A downlink failure occurs when *DSC* ≤ 0. As we discuss in [21] the failure time is 5.296 s if all the messages are blocked by the interference source. Indeed, this time actually does not depend on *N* since BS_PA_MFRMS also controls the frequency of the paging messages.

When a radio link failure occurs, the MSs will invoke their basic cell search procedure until the interference stops and they are reconnected to the cognitive BTS. Let us remark that no transmission will be emitted by the MSs during reconnection. However, if the interference does not disappear, the MSs will not be able to reconnect, and the BTS will be unaware since messages are not exchanged during reconnection.

We propose the following mechanism to minimize the impact of this problem: (i) force periodic communications from the MSs to the cognitive BTS and (ii) perform channel reselection if a given ratio of previously registered MSs do not transmit periodic messages. In GSM, periodic communications can be forced through the “Location Update” Procedure.

MS signaling in Mobility Management (MM) idle state is described in [33]. An MS in MM IDLE state initiates signaling (i) at incoming or outgoing call initiation; (ii) when timers T3211, T3213, or T3212 ex0pire; or (iii) when a new location area is entered. The BTS does not control cases (i) or (iii). Case (i) includes timers that trigger a “Location Update” Procedure. Among these timers, T3212 is used after termination of MM service or MM signaling, that is, to perform periodic updating. This timer is configured with values from 1 to 255/10 h [33]. Therefore, by setting the minimum value, the updating procedure will be performed each 6 minutes. If a given ratio (20% in our experiments) of MSs do not perform the “Location Update” Procedure, channel reselection is triggered as described above.

This clearly mitigates the problems related to persistent noise sources in the downlink channel. Note also that the signaling load associated with selecting such a short period is not significant, since the cognitive BTS will usually serve few MSs and the “Location Update” procedures of the MSs are asynchronous. Finally, the timeout value for T3212 is broadcast in the System Information Type 3 message on the BCCH, in the control channel description information element.

### 3.6. Channel Reselection Procedure

The cognitive BTS implementation is based on Asterisk [36], OpenBTS [22] and GNU Radio [37] open source software (see [28]), and on Ettus USRP hardware [23]. The USRP is a programmable USB SDR device. We modified OpenBTS to achieve the channel change capability we have designed for preventing possible interferences, by developing two different procedures to cater for the MM idle and dedicated modes of cell phones.

When the cell phone is in MM idle mode [32], GSM does not provide any procedures to command the MSs to change channels, so we modified the “Cell reselection” procedure to offer a better cell to the phones. Specifically, we modified the *System Information Type 2* message. The network sends this message through the BCCH to all phones within the cell with information about BCCH allocation in neighboring cells. Additionally, we publish the frequency of the channel to which we wish the BTS to jump, as if it were a neighbour cell. We inform the cell phones that they have to take measures of current and surrounding cells to detect the strongest signal. When the BTS switches to the new frequency the phones change to the new channel once it is detected, as it is the strongest signal from the neighboring cells listed in the he *System Information Type 2* message.

**Figure 7.**
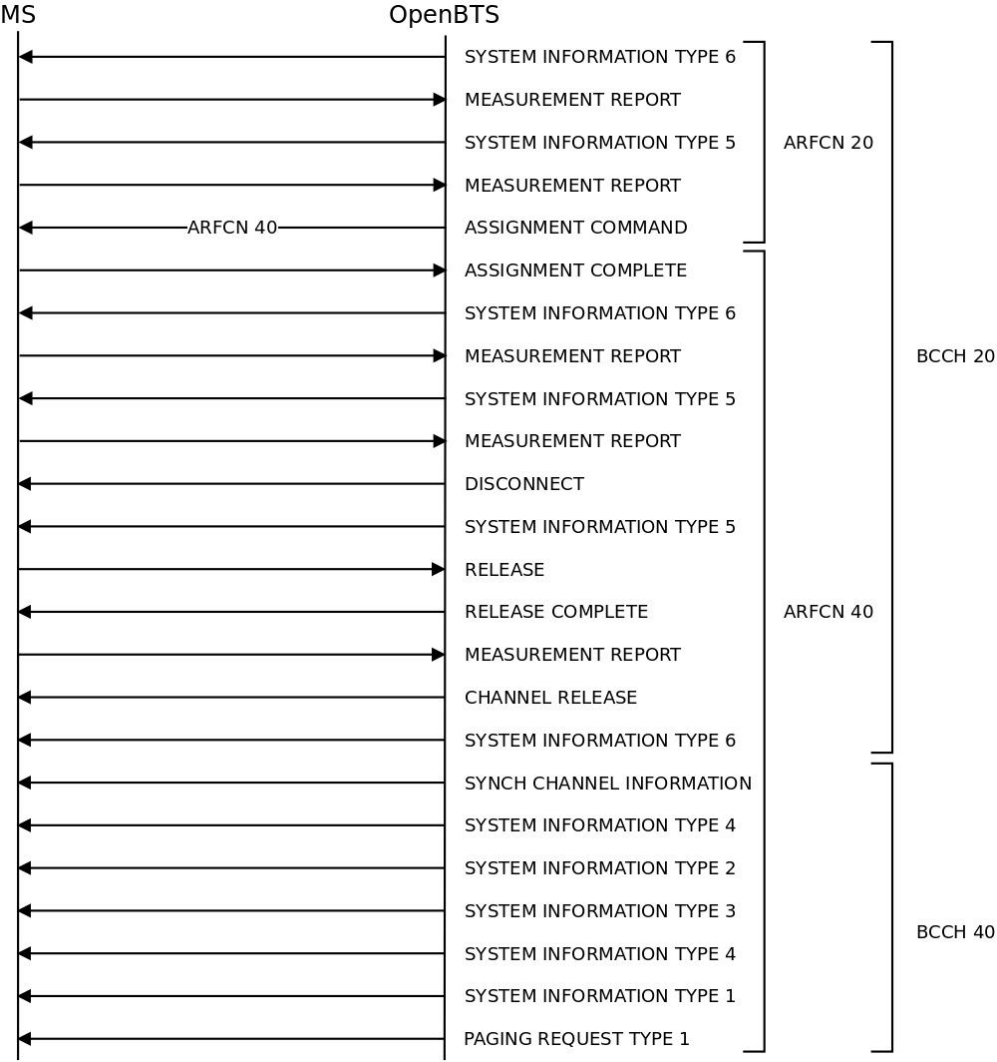
Layer 3 message exchange for channel change in dedicated mode.

When the phone is in dedicated mode [33], GSM provides the “Handover” and “Channel assignment” procedures. The “Handover” procedure cannot be implemented without two GSM carriers operating simultaneously. Therefore, we implemented the second procedure, and performed a successful channel change using the “Cell reselection” procedure, without any disruption. Figure 7 shows the complete process that corresponds to the diagram in Figure 3.

The BTS initiates the channel assignment procedure by sending an *Assignment Command* message to the MSs on the main signaling link. All other transmissions of signaling layer messages are suspended until the BTS effectively changes the channel and indicates that it has resumed operation. When the MSs receive the *Assignment Command* message, they initiate a local end release of link layer connections, disconnect the physical channels, switch to the assigned channel and initiate the establishment of new lower layer connections. The message contains the new channel configuration and a *starting time*, which is the time the MSs have to wait before accessing the new channel (this is necessary to give the cognitive BTS enough time to change to the new channel). Finally, the MS returns an *Assignment Complete* message, specifying a “normal event” cause to the network through the main Dedicated Control Channel (DCCH). Note that this procedure only allows the redefinition of the TCH for an ongoing communication; it does not inform about the broadcast control channel (BCCH) change. Therefore, the BTS must make the MSs aware of the main channel change. To do this, throughout the call, the BTS announces the BCCH allocation in the neighbouring cells in the *System Information Type 5* message. We thus modified that message to command the MSs to take measures in the new channel. This mechanism allows both the cognitive BTS and the MSs to change to the new channel without disruptions, avoiding interferences and degradation.

### 3.7. Cognitive BTS Additional Services: Real-Time Terminal Detection

As an added value, the interference avoidance mechanism also allows the detection of MSs, since they must emit messages during channel reselection. Let us define *T_D_* as the *detection time*, which should be low for the detection feature to work properly in case of emergency. *T_D_* depends on the state of the MS. In idle mode, it will depend on downlink channel signaling failure detection time plus the time it takes the MS to find the new carrier, as we discussed previously (see also [21]). According to our previous work [21] this time is less than 7.696 seconds, which is acceptable.

As previously explained, the channel assignment procedure is used when the cell phone is in dedicated mode. If so, once the MS has received the *Assignment Command*, it starts the channel change immediately, so *T_D_* will be much lower than in idle mode. In this case, *T_D_* corresponds to the time for the cognitive BTS to change the operating channel (which is hardware -and software- dependent) plus the time for the MS to process the message and to send the *Assignment Complete* response. The *starting time* parameter delivered with the *Assignment Command* can be used for the MSs to wait some time before launching the channel change process. This extra time allows the hardware to change the carrier frequency effectively. Since there are no disruptions in active conversations during channel change, *T_D_* is less than 50 ms (*i.e.*, the threshold for a user to perceive disruption in voice communications).

Therefore, when detecting a single MS, *T_D_* is satisfactorily low in all cases. In Section 4.5 we also study the case with multiple user terminals.

## 4. Validation Experiments

**Figure 8.**
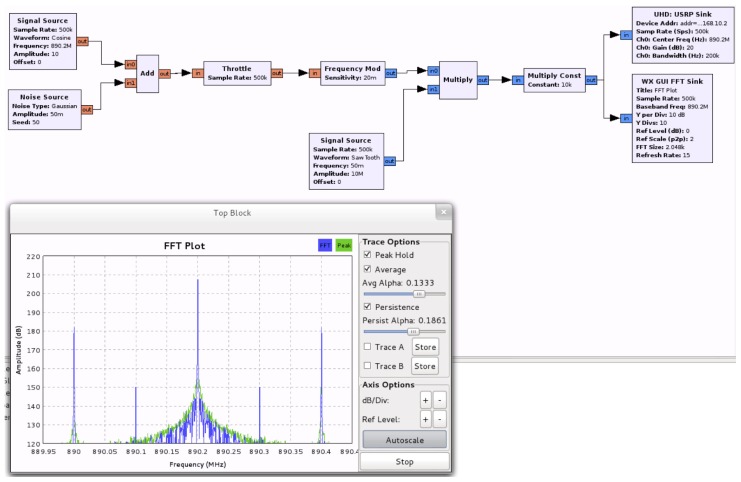
Jammer building blocks.

We tested our cognitive BTS system in two different setups. First, we checked all the control procedures described in the previous section and then we deployed the cognitive BTS in an indoor scenario. The main elements used in these experiments were:

- Our cognitive BTS built on a Linux laptop with an Ettus Research USRP, with two RFX900 front-ends with omnidirectional antennas, which we modified with an external 52 MHz clock to improve frequency precision. As previously said, the laptop ran GNU Radio, OpenBTS and Asterisk software.
- Commercial cellular phones: Nokia 6230 and Panasonic X400.
- Engineering test cellular phone: Ericsson SH888 TEMS unit (software version TMS600/4R1E, monitored with software TEMS GSM 900 1900/98.0.3.2).
- An FSH6 Rohde&Schwarz spectrum analyzer.
- A frequency-selective jammer based on a Linux computer and another Ettus Research USRP, also with two RFX900 front-ends and omnidirectional antennas. Figure 8 shows the building blocks of this system. This jammer emulated interferences with particular mobiles or with the BTS. Usually, in a real scenario, a cell is not fully affected by interferences, but only some of their components. This effect is emulated by disabling specific uplink/downlink channels, as corresponds to the situation in a large vehicle, such a vessel. Note again the versatility of SDR systems, which in this case served to configure auxiliary laboratory equipment.

### 4.1. Cognitive BTS Testbed

A full operative testbed with the main procedures in our solution was implemented. Its results are discussed next.

**Figure 9.**
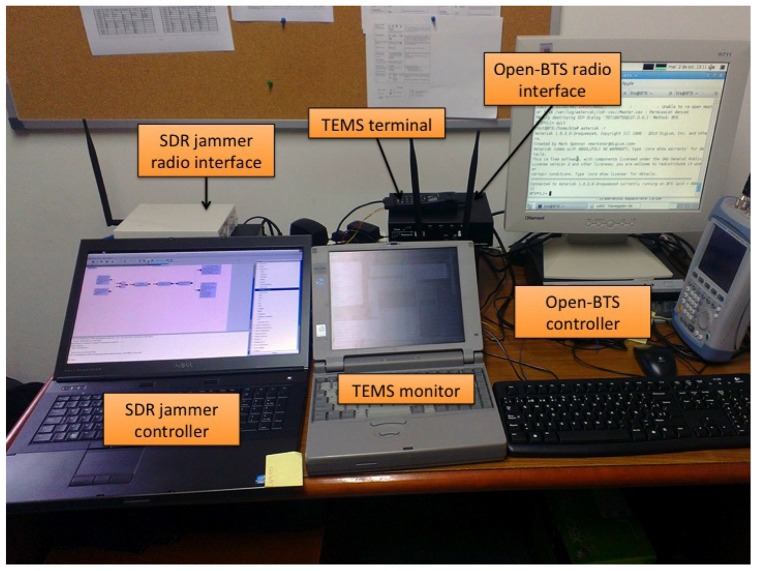
Experimental cognitive BTS setup.

**Figure 10.**
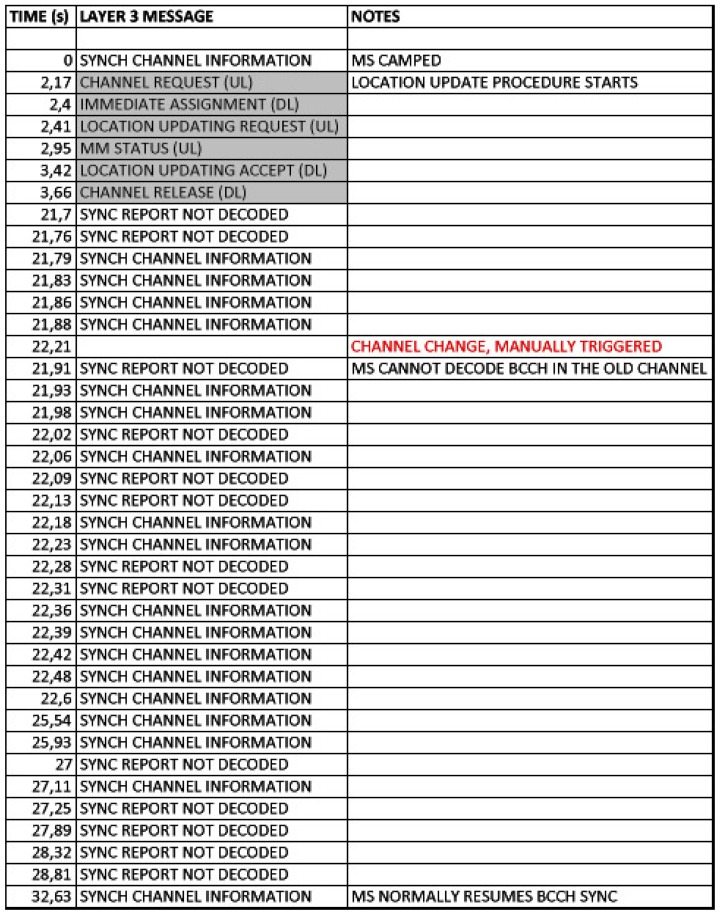
Idle mode—Normal cell reselection, Location Update procedure samples.

**Figure 11.**
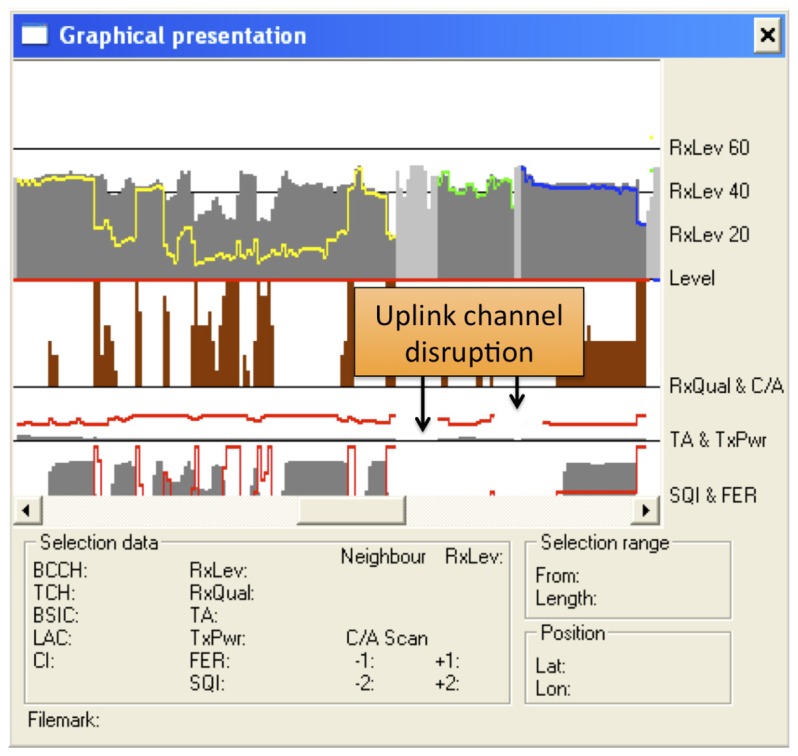
Uplink loss and cell reselection.

**Figure 12.**
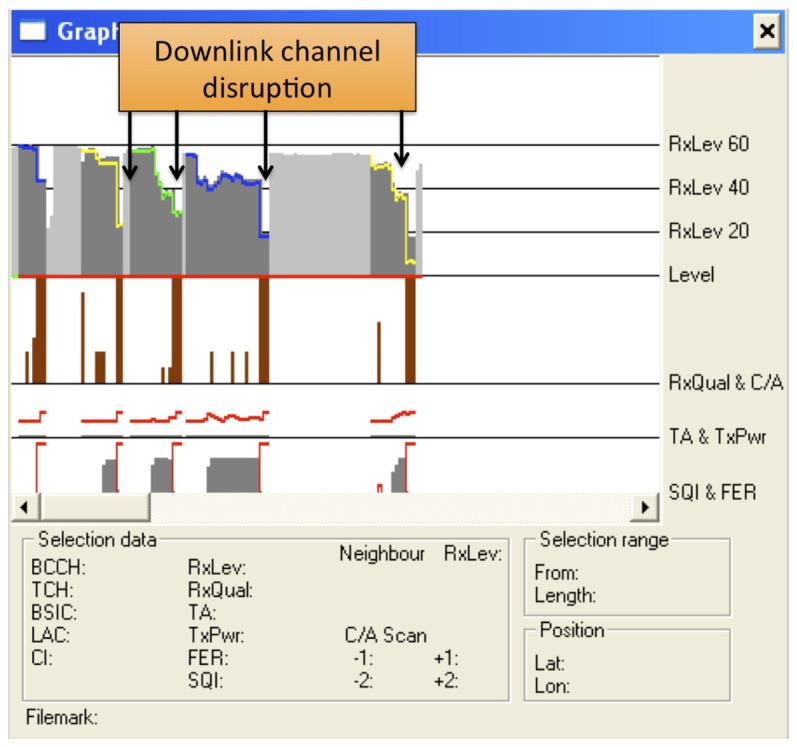
Downlink loss and cell reselection.

Figure 9 shows the main elements in our setup. The following experiments were performed:

1. **Connected mode—Normal cell reselection**. This experiment tested normal cell reselection with an ongoing call. A trace of the signaling involved has already been presented in Figure 7. Several tests were performed, with seamless changes of the cell channel in less than 570 ms in all cases. Speech communications continued fluently during the changes. Other tests with two simultaneous voice calls yielded the same results.
2. **Idle mode—Normal cell reselection**. This experiment tested cell reselection while the MSs were in idle mode. In this case, when the MS is aware of the channel change, it directly tunes to the new frequency without signaling exchange with the BTS. The maximum time measured in the experiments was 13.1 s. As an example of this procedure, Figure 10 shows how an MS first camps completely on a cell, which later changes its working channel. Once this occurs, the MS reselects that channel and continues to monitor broadcast and paging channels.
3. **Uplink loss and cell reselection**. This experiment tested the ability of the cognitive BTS to detect interference in the communication uplink. Obviously, this can only be detected when there are ongoing communications (otherwise, the MSs do not use the uplink). We emulated the effect of an interfering BTS nearby by activating our jammer in the corresponding part of the uplink spectrum. The jammer disabled any possible transmission from the MSs. Figure 8 shows the jammer tuned to 890.2 MHz, which is the uplink frequency of the channel with ARFCN #1.
  Figure 11 shows behavior of the MS. The upper part shows the received power in the downlink and the middle part shows the MS transmission power. In our experiment the jammer was activated twice at the instants shown in the figure. In both cases, the MSs stopped working, since they could not receive confirmation of their messages (both communication gaps can be clearly seen in the figure). Moreover, in each case, the cognitive BTS detected that call quality dropped below the critical level and issued a channel change. Note that channel frequencies are shown as different color lines (yellow, green and blue).
4. **Downlink loss and cell reselection**. Similarly to the previous case, this experiment checked the detection of communication problems in the downlink channel. Figure 12 shows the corresponding traces. The jammer was activated several times to force the cognitive BTS to perform a channel reselection. Each activation is indicated in the figure. It can be clearly seen how there was a sharp decline in the quality of the downlink channel, and how the channel changed each time.
5. **Downlink loss in idle mode**. This last experiment tested the recovery from a failure in the downlink channel when there were not ongoing communications. After the MSs camped on our cell, the jammer was activated to block the corresponding downlink carrier. After waiting for 6 minutes at most, we checked that the cognitive BTS noticed the absence of connection attempts from the MSs trying to initiate a “Location Update” procedure. Then, the cognitive BTS changed to a new channel, which the MSs eventually found and they camped again. In this case, the time from when the channel change took place until the MSs camped on the new cell was approximately one minute. Note that this is longer than in the normal cell reselection procedure in idle mode. This is as expected, since, when unable to decode the BCCH, the MSs initiate a full cell search procedure, which takes longer that the cell reselection procedure.

Some other problems related to the physical implementation of the cognitive BTS were addressed during these validation tests. We describe them in depth in the next sections.

### 4.2. Digital Compensation of Sensor Front-End Impairments

As said before, energy detection was based on a periodogram. The band of interest was divided into 4 MHz sub-bands that were analyzed sequentially using I/Q downconversion and sampling at the Nyquist rate of *fs* = 4 Msps (note that samples are performed in the complex domain). However, before applying this method, several impairments in the analog front-end of the SSM receiver had to be compensated, exploiting the flexibility of the SDR implementation. These impairments included a strong direct current (DC) component as well as a non-flat frequency response caused by the analog hardware components and filters. They were successfully addressed with two additional digital processing blocks: a DC canceling filter and an equalizer. Digitized signals at the output of an analog to digital converter (ADC) usually contain some DC bias. In our case it was significant, and we used the first-order IIR filter $H(z)=\frac{1-z^{-1}}{1-\rho z^{-1}}$ to remove it [38]. The pole value *ρ* = 0.996 was empirically adjusted for a sufficiently steep frequency response, preventing attenuation in the neighboring components. In order to uniformize the noise power spectral density across the sensed sub-band, the flatness of the frequency response was improved. A complex-valued linear phase FIR filter (equalizer) was designed for this purpose by generalizing the original design for real-valued filters in [39] to allow non-symmetric frequency responses as well as gain specifications at arbitrary frequency points (instead of uniformly spaced ones as in [39]).

**Figure 13.**
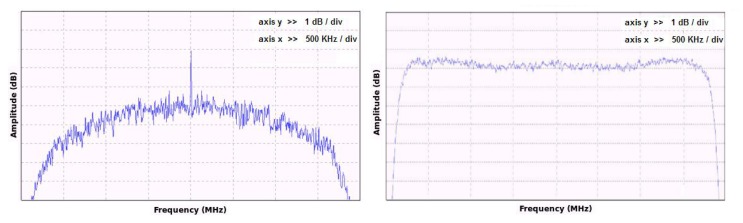
Frequency response before (left), and after equalization and DC cancellation.

Figure 13 shows the original non-flat frequency response of the system with the DC component and both defects corrected after the filtering. The steep fall in system response at the band edges is due to the transition band of the analog anti-aliasing filters. In the experimental prototype, the analysis was restricted to 3 MHz per sub-band (±1.5 MHz from center frequency) to avoid band-edge distortion. Thus, a total of $\frac{3\;\text{MHz}}{200\;\text{kHz}}=15\;\text{GSM}$ channels per sub-band could be simultaneously processed.

### 4.3. USRP Clock Problems

The clock of the USRP device is a low-cost 64 MHz crystal oscillator with 20 ppm precision. This caused two problems to OpenBTS: First, the clock frequency was not well adjusted to the GSM symbol rate, since it was not its multiple. In this case, the transceiver should implement a resampler to adjust to the right frequency, with the added burden of computational cost. Second, the USRP clock is not sufficiently stable and oscillator drifts could take the signal frequency outside the search range of mobile phones. This was especially relevant in the GSM 1.8 GHz band.

The solution to this problem was to install a specific 52 MHz clock with better precision. With it, the MSs were able to successfully camp on the cell without trouble and the computational load of the PC was greatly reduced. It was necessary to integrate the clock in the USRP motherboard and to change the OpenBTS software to disable frequency resampling.

**Figure 14.**
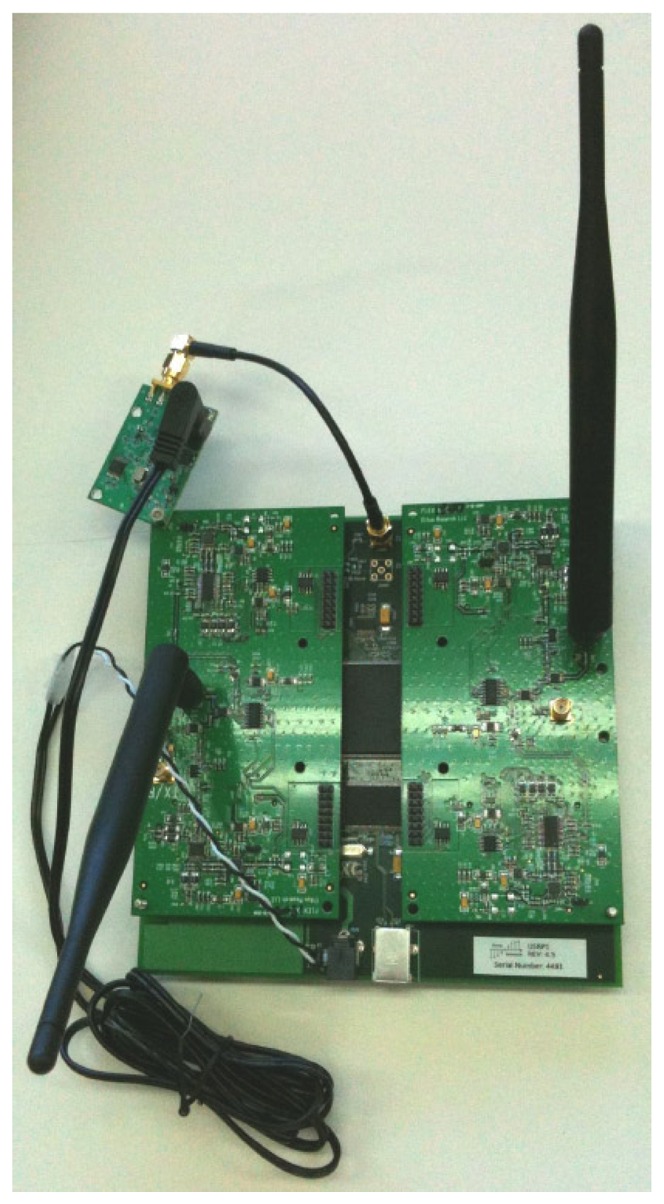
USRP board with auxiliary 52 MHz clock installed.

Figure 14 shows the USRP with the 52 MHz clock installed. The clock is based on the Temperature Compensated Crystal Oscillator (TCXO) Connor Winfield D53G and has a frequency stability of 0.5 ppm and a RMS phase jitter of close to 2 picoseconds.

### 4.4. Spectrum Sensing

Once the receiver frequency response was equalized in the initial off-line calibration step, the detection process could take place. Power levels were estimated based on the periodogram (squared magnitude of the FFT of the received signal) [40].

We used an FFT of length *N* = 1,024, with the possibility of averaging the periodograms over *P* ≥ 1 blocks. The estimated power level in channel *n* is given by averaging all the bins within the channel, *i.e.*,

(1) $${\text{PL}}_{n}=\frac{1}{N_{n}P}\sum_{p=1}^{P}\sum_{k\in A(n)}{\left|X_{p}\left(k\right)\right|}^{2}$$

where *Xp*(*k*) denotes the *N*-point FFT of the *p*-th block, *A*(*n*) is the set of frequency bins corresponding to channel *n*, and *N_n_* is the cardinality of *A*(*n*). Let the sensing time for a 3-MHz subband be *T_sensing_* = *N* · *P*/*fs*. Then, the total time to scan the whole 25-MHz bandwidth of the GSM900 downlink is

(2) $$T=\left\lceil \frac{25}{3}\right\rceil \left(T_{\text{sensing}}+T_{\text{settling}}\right)$$

where *T_settling_* is the settling time after the center frequency changes (milliseconds in our case). In spectrum sensing systems, the decision about the status (busy/idle) of a given downlink channel depends on the background noise level (NL) reference, which degrades performance randomly [41]. In order to overcome this problem, since some downlink channels were always idle, we estimated the NL as the lowest power level across all channels of the GSM900 downlink. The corresponding channel was automatically tagged as idle. The remaining channels were subject to statistical hypothesis tests, according to which channel *n* was busy if the ratio of the estimated power level *PL_n_* in that channel to the estimated NL exceeded a threshold.

The threshold was set for sufficiently small probabilities of false alarm (*P_FA_*) and missed detection (*P_MD_*) for signals that exceeded the NL by 9 dB (remember that this is the minimum *C*/*I* level required for GSM communications [42]). Assuming that the measured power levels follow a Gaussian distribution and that the background noise is Gaussian and uniform across the whole bandwidth, *P_FA_* and *P_MD_* can be derived in closed form.

**Figure 15.**
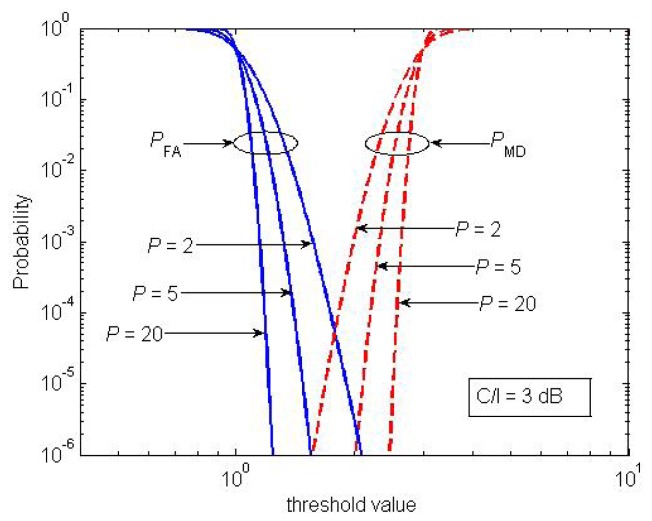
Detection performance (*N* = 1,024, *C*/*I* = 3 *dB*).

Figure 15 plots these levels as a function of the threshold for *C*/*I* = 3 dB and for different values of *P*. It shows how *P* = 5 blocks sufficed to set a threshold that simultaneously achieved *P_FA_* = 10^−6^ and *P_MD_* = 10^−6^. Note that a failed detection resulted in a missed carrier, whereas a false positive meant that an empty channel was misidentified as busy. With *P* = 5, the whole 25-MHz bandwidth was scanned in less than 0.5 seconds.

### 4.5. Detection Tests for Multiple Terminals

The detection time estimation in Section 3.6 is valid only in cases where there is a single MS. It was therefore necessary to study detection scalability as the number of terminals grows. We consider that a number between 5 and 100 terminals is realistic. In this situation another important contribution to total detection time must be taken into account: the worst-case contention time *T_C_* for *N* MSs, due to the contention mechanism used in the random access channel (RACH), for the MSs to request a dedicated channel during channel changes [33]. This time is the time that it takes for the last MS to obtain an empty slot in the RACH.

In short, all the terminals contend for an empty slot in the RACH in order to transmit a *Request Channel* message. They transmit the message in a single slot and then wait for the response. When a collision occurs (when several MSs transmit their messages in the same slot), the affected terminals do not receive any response and a uniform distribution is used to determine the slot for the next attempt. This distribution depends on the *T_x_*_−_*_integer_* parameter, which is broadcast on the BCCH and on the Common Control Channel (CCCH) configuration [33].

We needed to estimate the average *T_C_* in order to test the suitability of our proposal in a real emergency. Consequently, we simulated the behavior of *N* MSs requesting a dedicated channel, with a *T_x_*_−_*_integer_* value of 14, assuming a combined CCCH, which resulted in a random uniform distribution for the number of slots between attempts in [41,54]. We simplified the model by eliminating the maximum number of attempts, assuming that all the MSs would initiate the requesting process at the same time, which is realistic in our scenario because the forced channel change triggers this process for all active MSs.

We evaluated *T_C_* by increasing *N* in [5,100] in steps of 5. For each step, we averaged samples until a confidence interval with a tolerance of 1% was achieved with a 99% confidence level at least. The number of simulations needed to satisfy these requirements was determined using the Batch Means method [43], since simulation samples exhibited low term correlation.

**Figure 16.**
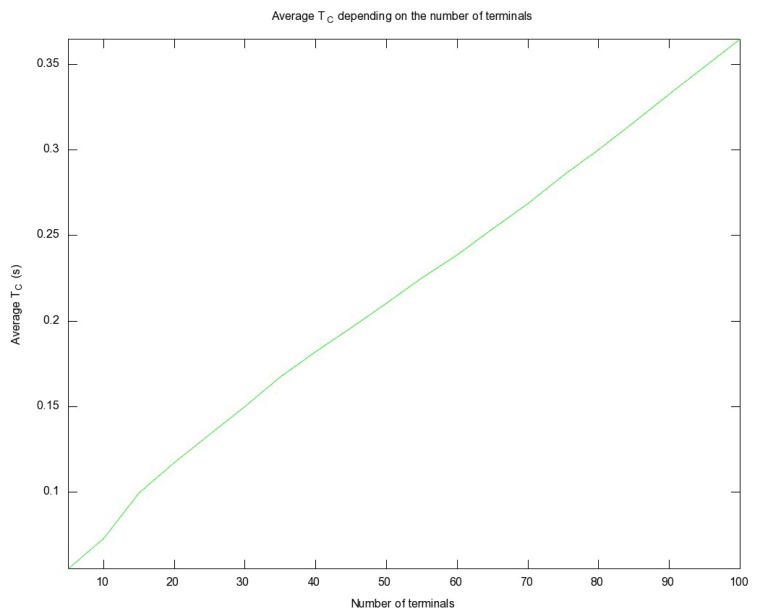
Average *T_C_* for *N* terminals.

Figure 16 shows the average *T_C_* depending on the number of MSs in the scenario. The standard deviation of *T_C_* varied between 0.01 and 0.02 seconds (corresponding to 10 and 100 terminals, respectively). The results indicate that, as expected, the average contention time grows proportionally to the number of MSs, but it is reasonably low in the application scenario. This confirms the hypothesis that our proposal would satisfy the requirements in emergency situations, since the contribution of *T_C_* to total response time is negligible compared with *T_D_*.

## 5. Conclusions

We have proposed a mobile cognitive BTS with spectrum sensing capabilities that takes advantage of the flexibility of SDR technology to deploy added-value services such as terminal detection in case of emergencies. The cognitive BTS provides communication services inside moving vehicles, avoiding interference in real-time. We have developed and tested the most critical parts of our proposal, demonstrating their correct behavior and the validity of our approach.

As future work, we plan to incorporate new services based on intelligent spectrum sensing, which are not available in conventional cellular networks.
